# Combination of Risks of BMI and Health-Related Lifestyles on Kidney Function in the Prediabetic Japanese Population: A Prospective Cohort Study

**DOI:** 10.3390/ijerph20075338

**Published:** 2023-03-30

**Authors:** Jou-Yin Chen, Shiqi Deng, Yukiko Wagatsuma

**Affiliations:** 1 Department of Clinical Trials and Clinical Epidemiology, Graduate School of Comprehensive Human Sciences, University of Tsukuba, Tsukuba 305-8575, Japan; 2 Department of Clinical Trials and Clinical Epidemiology, Faculty of Medicine, University of Tsukuba, Tsukuba 305-8575, Japan

**Keywords:** prediabetes, progression of chronic kidney disease, lifestyles, overweight

## Abstract

Diabetic overweight patients are more likely to show the progression of kidney damage than the general population. The majority of people in the early stages of kidney damage do not recognize the importance of risk modification, mainly due to the asymptomatic nature of the disease. This study aimed to examine specific risk combinations of lifestyle and BMI regarding the deterioration of kidney function and to explore whether there are gender-based differences among the prediabetic population. Prediabetic participants with normal kidney function were identified via annual health examination from April 2016 to March 2019. The information on health status and lifestyle was collected at enrollment. The study subjects were followed until March 2021 to observe the progression of kidney damage. There were 2241 participants enrolled in this study. Smoking (HR = 3.5, *p* < 0.001), eating snacks (HR = 3.2, *p* < 0.001), not engaging in regular exercise (HR = 2.9, *p* < 0.001), and not having adequate sleep (HR = 3.0, *p* < 0.001) showed accelerated risks for kidney damage progression among the prediabetic population in males. These lifestyle effects were not observed in females. In conclusion, risk-based modification of lifestyle behavior is important to prevent kidney function damage among the overweight prediabetic population in males.

## 1. Introduction

Chronic kidney disease (CKD) refers to the gradual loss of kidney function. In Japan, the prevalence of CKD has increased in the general Japanese population over the last three decades [1]. The American National Kidney Foundation classified chronic kidney disease into five stages according to the estimated glomerular filtration rate (eGFR) [2]. End-stage renal disease (ESRD) refers to stage 5 of CKD with an eGFR of less than 15 mL/min/1.73 m^2^, which is considered an irreversible reduction in kidney function. In addition, CKD is often associated with diabetes. Diabetic kidney disease (DKD) is one of the chronic complications of diabetes. In Japan, the prevalence of DKD is 52% among diabetic patients, increasing the burden of healthcare costs [3,4]. Moreover, DKD patients are more likely to develop ESRD than nondiabetic populations [5]. Several studies have demonstrated that kidney damage increases mortality in the diabetic population compared to the general population [6,7].

Even if CKD is progressive and irreversible, it can be effectively managed before it advances to the later stages. The incidence and progression of CKD are commonly found in the older population or those with a diabetes duration of more than 10 years [8]. To promote a healthy population and reduce the medical burden, it is critical to make every effort to prevent the deterioration of the kidneys in the early stages in the diabetic population [9]. There are no obvious symptoms in the early stage of CKD, which may lead to the management of kidney function status being neglected. Healthy lifestyles such as an appropriate diet, physical activity, and managing body weight have been reported to be positively related to CKD management [10]. However, studies exploring lifestyle in the early stages of CKD among the prediabetic population are relatively limited [11,12,13].

Being overweight is a common complication in the diabetic population. In Japan, the prevalence of overweight patients (BMI ≥ 25) among the diabetic population was around 50% in 2012, which means that one in two diabetic patients was overweight; in addition, the prevalence is increasing [14]. Even though the diabetic overweight population is increasing, studies investigating lifestyle in relation to kidney damage among overweight prediabetic population are lacking. Furthermore, sex differences should be considered due to the differences in physiology [15,16]. Therefore, this prospective cohort study aimed to examine specific risk combinations of lifestyle and BMI in relation to the deterioration of kidney function and to explore whether there are gender-based differences in kidney damage among the prediabetic population.

## 2. Materials and Methods 

### 2.1. Study Subjects and Data Extraction

Study subjects were recruited from an ongoing cohort study project, aiming to contribute health information and improve the health status in the community. The participants were invited to attend an annual health examination at a regional hospital in Mito or its outreach service sites in Japan. Approximately 5000 participants attended the health examinations annually. Trained research staff went to the health check-up center to collect health information monthly. After data collection, data cleaning was implemented by a trained data manager. This study was approved by the research ethics committee of the Faculty of Medicine at the University of Tsukuba (approval number: 1000). It was carried out in accordance with the Declaration of Helsinki. Written informed consent was obtained from each participant.

### 2.2. Study Design

This was a prospective cohort study. Prediabetic subjects with normal kidney function who underwent health examinations from April 2016 to March 2019 were included. The first enrollment date of each subject during this period was set as the baseline point. Demography, lifestyle factors, anthropometric variables, and clinical values were collected at the baseline point. The subjects were followed to observe the progression of kidney damage until March 2021. 

### 2.3. Measures and Definitions

This study included the prediabetic population with normal kidney function. Prediabetes were defined as HbA1c greater than 5.6% or fasting blood sugar greater than 100 mg/dL according to the Japanese Clinical Practice Guideline for Diabetes in 2019 [17]. In addition, subjects who were taking anti-diabetic medication, and those who had a eGFR below 60 mL/min/1.73 m^2^ or had proteinuria at baseline were excluded. 

The outcome of this study was the progression of kidney damage, defined as an eGFR of less than 60 mL/min/1.73 m^2^ or having proteinuria, which was measured using a urine protein dipstick (Uropaperα III^®^, Eiken Chemical Inc., Tokyo, Japan) during the follow-up period. Proteinuria was defined as protein of more than 30 mg/dL in urine. Those participants who had not experienced any progression in kidney damage by the endpoint of the study were defined as censor data. The health examination date of the outcome was considered the event timepoint for the survival analysis.

Anthropometric information such as body weight and height was collected using the Tanita DC250 (TANITA Co., Tokyo, Japan). Body mass index (BMI) was calculated as weight/height^2^. Due to the low proportion of obese subjects, i.e., those whose BMI was more than 30 (8%), this study categorized the BMI groups as being in the normal range (BMI < 25) and overweight (BMI ≥ 25) for males. A lower cut-off of BMI ≥ 23 for overweight patients was used for females, as suggested in previous studies on the Asian population [18,19]. Blood pressure measurements were performed by professional medical staff at the regional hospital or outreach centers. Blood samples are collected and biochemical tests such as high-density lipoprotein, low-density lipoprotein, triglycerides, HbA1c, fasting plasma glucose, and creatinine were measured at the clinical laboratory in the regional hospital. eGFR was calculated using the formula: eGFR = 194 × serum creatinine × (−1.094) × age × (−0.287) × 0.739 (if female) [20]. 

Hypertension was defined as systolic blood pressure ≥ 140 mmHg or diastolic blood pressure ≥ 90 mmHg, and dyslipidemia was defined as triglycerides ≥ 150 mg/dL or low-density lipoprotein ≥ 140 mg/dL or high-density lipoprotein < 40 mg/dL [21].

Lifestyle factors were collected through a self-reported questionnaire of 22 items (a set of standardized questions for chronic disease control specified by the Ministry of Health and Welfare, Japan). The full questionnaire is shown in Supplemental File S1. This study selected some questions related to the study aim: smoking status (smoking in the last one month); alcohol consumption frequency (every day, sometimes, or rarely); exercise of more than 30 min per session, more than two times per week, and continuously for more than 1 year; daily walking or performing other physical activity equal to walking for more than 1 h per day; having snacks or sweet drinks in addition to three meals regularly; skipping breakfast more than three times per week; getting adequate sleep or not.

### 2.4. Statistical Analysis

Baseline demographics, lifestyle factors, anthropometric variables, and clinical values are presented as the mean values with standard deviations for continuous variables, and counts with percentages for categorical variables. The differences in characteristics according to sex at baseline were assessed using *t*-tests for continuous variables and chi-square tests for categorical variables. The dates that participants met the prediabetes definition were defined as the first timepoints, and the end timepoints were set as the date when participants were noted to have experienced a progression in kidney damage. The follow-up period for each participant was different. Cox proportional hazard models were used to assess whether each BMI category combined with different lifestyle factors would predict the occurrence of progression of kidney damage. All statistical analyses were conducted using SAS version 9.4, with a 0.05 significance level at a 95% confidence interval.

## 3. Results 

**Figure 1:**
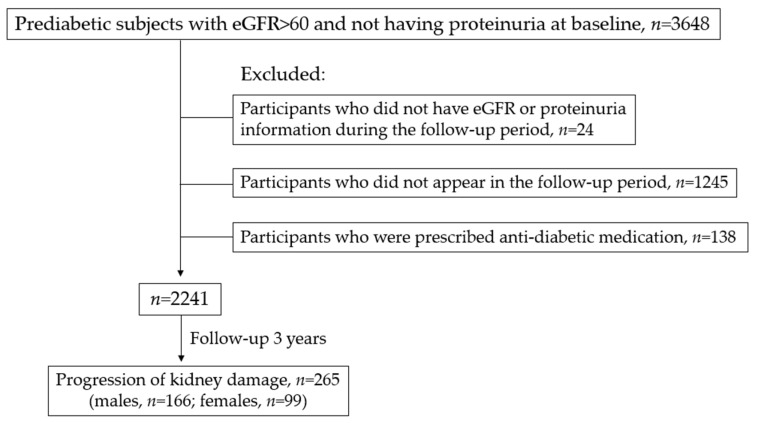
Study flow chart.

This study’s flow chart is presented in Figure 1. There were 3648 prediabetic subjects with normal kidney function enrolled from April 2016 to March 2019. After excluding individuals who did not have eGFR or urine protein information during the follow-up period (*n* = 24), those who did not appear in the follow-up period (*n* = 1245), and those who were prescribed anti-diabetic medication (*n* = 138), 2241 subjects were included in this study. The mean duration of the follow-up period was 32.5 months (SD = 13.7 months) and the median was 35.8 months. During the follow-up period, 166 (13.5%) males and 99 (9.7%) females developed progression of kidney damage, respectively. 

Table 1 shows the demographics, comorbidities, clinical values, and lifestyle characteristics at baseline stratified by sex. The mean age was 52.7 years. Most of the participants were middle-aged (40–65 years old). There were 1226 (54.7%) males. The women were significantly older than the men (mean age of 50.4 vs. 55.4 years for males and females, respectively; *p* < 0.001). 

The mean eGFR at baseline was 77.4 mL/min/1.73 m^2^, representing a mildly decreased kidney function (G2 of CKD stage). The men had significantly higher mean eGFR values than the women at baseline (*p* = 0.002). Meanwhile, the men had significantly higher BMI values (24.9 ± 3.8 vs. 23.2 ± 3.9 kg/m^2^, *p* < 0.001), higher proportions of hypertension (37.0% vs. 30.9%, *p* = 0.003) and dyslipidemia (49.7% vs. 38.6%, *p* < 0.001), and higher uric acid (6.0 ± 1.2 vs. 4.5 ± 1.0 mg/dL, *p* < 0.001) than the women. In total, 36% of the study subjects were overweight or obese. The average HbA1c was 5.9%. Regarding lifestyle factors, the men had a significantly higher proportion of smoking, alcohol consumption, and skipping breakfast habits than the women; however, the men also exercised more and slept more adequately than the women. 

**Figure 2:**
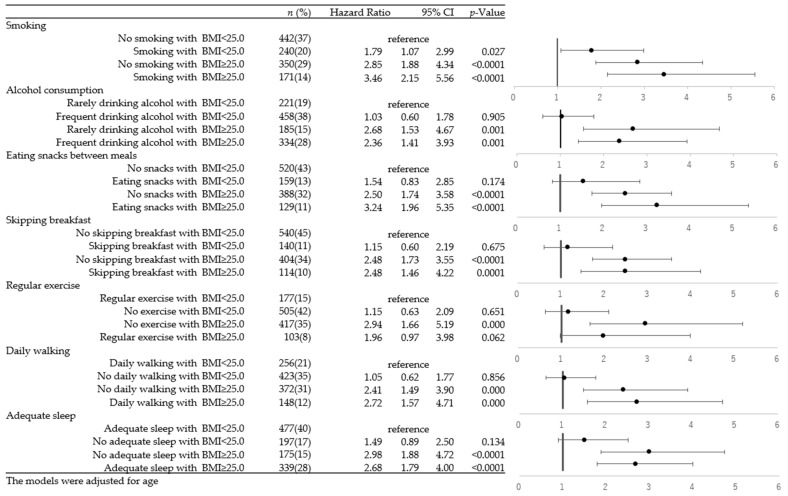
Cox proportional hazard model of the combination of BMI and lifestyles and the progression of kidney damage among males.

**Figure 3:**
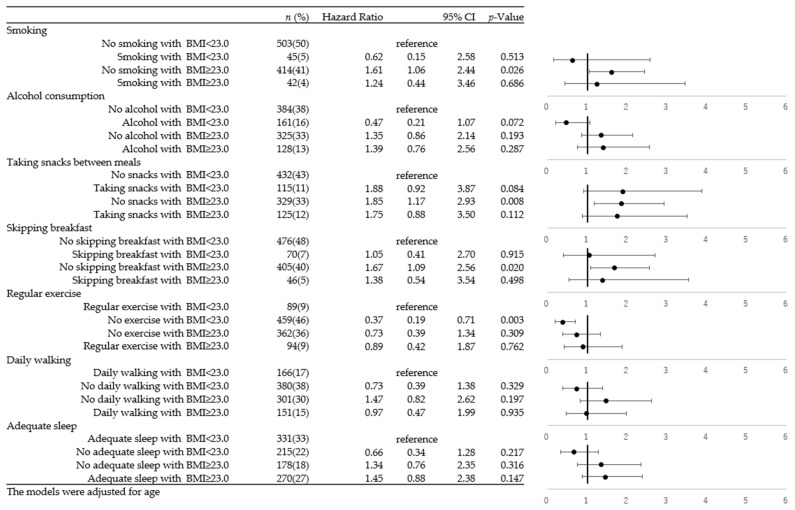
Cox proportional hazard model of the combination of BMI and lifestyles and the progression of kidney damage among females.

The risk of the combination of lifestyle factors and BMI levels with the progression of kidney damage among overall subjects is presented in supplemental Figure S1. Figure 2 and Figure 3 show the sex stratification for the combination of lifestyle factors and BMI levels with the risk of progression of kidney damage for males and females, respectively. Regardless of any lifestyle, the groups with a BMI of more than 25 demonstrated a significantly higher risk of the progression of kidney damage than the groups with a BMI of less than 25. For the lifestyles of smoking, alcohol consumption, eating snacks between meals, and skipping breakfast, the combination of not having these lifestyles and having a BMI of less than 25 kg/m^2^ was set as the reference group. 

In Figure 2, which summarizes the values for males, one can observe that smoking, occasionally drinking alcohol, and eating snacks between meals all show associations with a higher risk of the progression of kidney damage among subjects with a BMI of more than 25 (smoking with a higher BMI, HR = 3.46; no smoking with a higher BMI, HR = 2.85; no alcohol with a higher BMI, HR = 2.68; alcohol with a higher BMI, HR = 2.36; eating snacks between meals with a higher BMI, HR = 3.24; not eating snacks between meals with a higher BMI, HR = 2.50). For the lifestyle habits of regular exercise, daily walking, and adequate sleep, the combination of having these lifestyle habits with a BMI of less than 25 kg/m^2^ was set as the reference group. Not having regular exercise, daily walking, or adequate sleep were shown to be associated with higher risk of the progression of kidney damage among the overweight prediabetic population (not engaging in exercise with a higher BMI, HR = 2.94; daily walking with a higher BMI, HR = 2.72; no daily walking with a higher BMI, HR = 2.41; inadequate sleep with a higher BMI, HR = 2.98; adequate sleep with a higher BMI, HR = 2.68).

In Figure 3, which summarizes the values for females, being overweight is shown to have an effect in the progression of kidney damage, rather than lifestyle behaviors. A higher BMI was shown to be associated with a significantly higher risk of the progression of kidney damage in female subjects who did not smoke, did not eat snacks between meals, and did not skip breakfast (no smoking with a higher BMI, HR = 1.61; no snacks with a higher BMI, HR = 1.85; no skipping breakfast with a higher BMI, HR = 1.67). Lifestyle behaviors did not show aggravated risk of the progression of kidney damage among overweight prediabetic subjects in females. The results for females defined as overweight with a BMI greater than 25 are presented in supplemental Figure S2. Similar findings were observed.

## 4. Discussion

This study explored the combination of risks from health-related lifestyle habits and BMI in relation to kidney function among prediabetic subjects. We found that the lifestyle behaviors of smoking, occasionally drinking alcohol, eating snacks between meals, not engaging in exercise, daily walking, and not having adequate sleep are associated with a higher risk of kidney damage progression among the overweight prediabetic males. As for females, being overweight was shown to have an effect on the progression of kidney damage, rather than lifestyle behaviors. 

A significantly increased risk of kidney damage related to lifestyle habits was found in the overweight prediabetic population, but not in those subjects who had a BMI of less than 25. This may be due to the synergistic effect of lifestyle and being overweight contributing to the progression of kidney damage. A higher BMI has been observed to promote kidney damage by aggravating metabolic syndromes such as hypertension, insulin resistance, and glomerular hyperfiltration [22]. The association between lifestyle habits and increased risk of kidney damage has also been reported in previous studies. Smoking aggravates kidney failure through the mechanism of sustained sympathetic activity or oxidative stress [23]; snacking is often accompanied by a high intake of calories, and high levels of salt or sugar intake have been found to cause harm to kidney function [24,25]; regular breakfast consumption is suggested to prevent kidney failure by decreasing the negative effect on glucose and insulin metabolism [26]. Moreover, diabetes is often associated with several comorbidities that elevate the risk of kidney damage [27]. The combination of these negative conditions may accelerate the progression of kidney damage.

Previous studies have mentioned that drinking alcohol is a “double-edged sword” for kidney function. Excessive drinking has been found to lead to the progression of kidney damage due to the induction of reactive oxygen species, which is linked to oxidative stress in the kidneys. However, studies have also found that moderate alcohol consumption can increase insulin sensitivity, which can benefit kidney function [28]. In this study, compared to the frequent alcohol drinkers, occasional alcohol drinkers showed higher risk of kidney damage progression among overweight prediabetic males. However, since heavy alcohol consumption has also been proven to be associated with the progression of kidney damage [29], the amount of alcohol consumption should be considered in further studies. One past study suggested that daily walking steps of around 7000–12,000 are related to a high quality of life among CKD patients [30]. However, in this study, a daily walking habit showed a slightly higher risk of the progression of kidney damage in males. Compared to rigorous regular exercise, which is defined as more than 30 min per session, more than two times per week, and continuing for more than one year, daily walking is a relatively mild physical exercise habit and higher rates of daily walking were observed among the elderly population. Biases may occur in a population that is sicker or older. Therefore, we believe that daily walking benefits kidney function.

Regardless of lifestyle habits, groups with a higher BMI had a significantly higher risk of kidney damage progression. In previous studies, obesity has been reported to be a risk factor for CKD [31,32], and it also accelerates the progression of renal function damage and increases the risk of developing end-stage renal disease in the diabetic population [33]. The biological mechanism of obesity-related kidney failure includes hemodynamic changes, adipose tissue increases, and insulin resistance pathways. The modulation of adipokines may cause inflammation and oxidative stress, as well as further injury to the kidney. Activation of the sympathetic nervous system may increase tubular sodium reabsorption and may cause glomerular hyperfiltration, finally leading to kidney injury [27]. The Japanese population is known to have a higher tendency to accumulate ectopic lipid despite having a relatively lower BMI than other ethnics. This could be due to a lower capacity for subcutaneous fat storage, leading to the excess lipid being stored in other areas of the body. It can lead to metabolic disturbances, including insulin resistance and impaired glucose tolerance [34]. 

Sex differences were found to exist when we explored the combination of BMI and lifestyle effects for kidney damage progression. Among males, the lifestyle habits of smoking, occasional alcohol consumption, eating snacks between meals, not engaging in regular exercise, daily walking, and having inadequate sleep showed associations with accelerated risk of kidney damage progression in the overweight prediabetic population. However, a significant effect of lifestyle habits on kidney damage progression was not found in females, in whom being overweight was shown to have an effect on the progression of kidney damage, rather than this being affected by lifestyle behaviors. Considering that Japanese women tend to be thinner, BMI values of more than 23 were used for the definition of overweight for females in this study [18,19]. The results were similar to those for which the cut-off point of 25 was used for BMI. Previous studies have indicated that health consciousness differs substantially by sex. In some studies, females have been reported to avoid unhealthy diets, and to have healthier lifestyle behaviors such as regular exercise or adequate sleep [35,36]. In addition, due to the lower percentage of overweight subjects in the female cohort, the power to detect differences was much lower in females according to various lifestyle factors. Another possible explanation for sex differences could be the distinction in physiology. Women have slower progression of kidney damage than men due to the protection of estrogen in the premenopausal status [37]. In this study, males had significantly higher eGFR levels than females at baseline; however, after the follow-up period, males progressed more than females. Longer follow-up periods might be required for investigating the differences in kidney function change between the sexes.

This study aimed to provide health-related information and promote good health status in the community. The strength of this study is the large number of study subjects with a stable return rate. Furthermore, it was a longitudinal study that measured the occurrence of the progression of kidney damage to make a causal inference. There are some limitations in this study. First, participants attending the annual health examination may have a potential selection bias due to health consciousness. Second, biases exist since the lifestyle information was collected using self-reported questionnaires. Since people tend to report healthier lifestyles, the results may be underestimated. Finally, since this study only included patients without kidney damage, the follow-up period may not have been long enough to observe the outcome events in this population.

## 5. Conclusions

Risk-based modification of lifestyle behaviors is important to prevent kidney function damage among overweight prediabetic males. Further studies are required to examine gender-based differences in kidney function damage and lifestyle behaviors.

## Figures and Tables

**Table 1:** Baseline characteristics of the study subjects.

	Total, *n* = 2241	Male, *n* = 1226	Female, *n* = 1015	*p*-Value
Demographic				
Age, years	52.7 ± 13.3	50.4 ± 13.6	55.4 ± 12.2	<0.001
Age group				<0.001
<40 years	391 (17.5%)	287 (23.4%)	104 (10.3%)	
40–65 years	1492 (66.5%)	780 (63.6%)	712 (70.1%)	
>65 years	358 (16.0%)	159 (13.0%)	199 (19.6%)	
Comorbidities and clinical values				
Baseline eGFR	77.4 ± 12.0	78.1 ± 12.0	76.6 ± 12.0	0.002
BMI	24.1 ± 3.9	24.9 ± 3.8	23.2 ± 3.9	<0.001
BMI category				<0.001
≥30	167 (7.5%)	111 (9.1%)	56 (5.5%)	
25–29	639 (28.5%)	416 (33.9%)	223 (22.0%)	
<25	1435 (64.0%)	699 (57.0%)	736 (72.5%)	
Hypertension				0.003
Yes	767 (34.2%)	453 (37.0%)	314 (30.9%)	
No	1474 (65.8%)	773 (63.1%)	701 (69.1%)	
Dyslipidemia				<0.001
Yes	1001 (44.7%)	609 (49.7%)	392 (38.6%)	
No	1240 (55.3%)	617 (50.3%)	623 (61.4%)	
Uric acid ^1^	5.3 ± 1.4	6.0 ± 1.2	4.5 ± 1.0	<0.001
Hemoglobin A1c ^2^	5.9 ± 0.5	5.9 ± 0.6	5.9 ± 0.4	0.857
Lifestyle factors				
Smoking ^3^				<0.001
Yes	498 (22.6%)	411 (34.2%)	87 (8.7%)	
No	1709 (77.4%)	792 (65.8%)	917 (91.3%)	
Regular exercise ^4^				0.004
Yes	463 (21.0%)	280 (23.3%)	183 (18.2%)	
No	1743 (79.0%)	922 (76.7%)	821 (81.8%)	
Alcohol consumption ^5^				<0.001
Everyday	474 (21.6%)	404 (33.7%)	70 (7.0%)	
Sometimes	607 (27.6%)	388 (32.4%)	219 (21.9%)	
Rarely	1115 (50.8%)	406 (33.9%)	709 (71.0%)	
Daily walking ^6^				0.337
Yes	721 (32.8%)	404 (33.7%)	317 (31.8%)	
No	1476 (67.2%)	795 (66.3%)	681 (68.2%)	
Eating snacks ^6^				0.955
Yes	528 (24.0%)	288 (24.1%)	240 (24.0%)	
No	1669 (76.0%)	908 (75.9%)	761 (76.0%)	
Skipping breakfast ^7^				<0.001
Yes	370 (16.9%)	254 (21.2%)	116 (11.6%)	
No	1825 (83.1%)	944 (78.8%)	881 (88.4%)	
Adequate sleep ^8^				<0.001
Yes	1417 (65.0%)	816 (68.7%)	601 (60.5%)	
No	765 (35.0%)	372 (31.3%)	393 (39.5%)	
Kidney damage progression				0.005
Yes	265 (11.8%)	166 (13.5%)	99 (9.7%)	
No	1976 (88.2%)	1060 (86.5%)	916 (90.3%)	

^1^ Missing, *n* = 418; ^2^ missing, *n* = 134; ^3^ missing, *n* = 34; ^4^ missing, *n* = 35; ^5^ missing, *n* = 45; ^6^ missing, *n* = 44; ^7^ missing, *n* = 46; ^8^ missing, *n* = 59.

## Data Availability

The data generated or analyzed during this study are available from the corresponding author upon request.

## Supplementary Materials

The following supporting information can be downloaded at: https://www.mdpi.com/article/10.3390/ijerph20075338/s1, File S1: Lifestyle questionnaire; Figure S1:Cox Proportional Hazard Model of the combination of BMI and lifestyles and the progression in kidney damage among overall subjects; Figure S2: Cox Proportional Hazard Model of the combination of BMI and lifestyles and the progression in kidney damage with a definition of overweight/obese as BMI ≥ 25 among females.
