# Supplementary material for: Combination of Risks of BMI and Health-Related Lifestyles on Kidney Function in the Prediabetic Japanese Population: A Prospective Cohort Study
Source: Int J Environ Res Public Health. 2023 Mar 30;20(7):5338. doi: 10.3390/ijerph20075338 (PMC10094487; doi:10.3390/ijerph20075338)

Supplemental File-S1. Lifestyle questionnaire.

1. Q: Are you currently using an anti-hypertensive drug?
   A: Yes/ No
2. Q: Are you currently using insulin injection or an antidiabetic (hypoglycemic) drug?
   A: Yes/ No
3. Q: Are you currently using an anti-cholesteremic agent?
   A: Yes/ No
4. Q: Have you ever been diagnosed as having a stroke (cerebral hemorrhage or infarction) by a physician, or had medical treatment?
   A: Yes/ No
5. Q: Have you ever been diagnosed as having heart disease (angina pectoris or myocardial infarction) by a physician, or had medical treatment?
   A: Yes/ No
6. Q: Have you ever been diagnosed as having chronic renal failure by a physician, or got artificial dialysis?
   A: Yes/ No
7. Q: Have you ever been diagnosed as having anaemia?
   A: Yes/ No
8. Q: Have you smoked in the last month?
   A: Yes/ No
9. Q: Have you put on weight by 10kg since your 20s?
   A: Yes/ No
10. Q: Have you exercised more than 30 minutes per 1 time, more than 2 times per week, more than 1 year?
    A: Yes/ No
11. Q: Do you walk daily or do other physical activity equal to walking more than 1 hour per day?
    A: Yes/ No
12. Q: Do you walk faster than those in same age with you?
    A: Yes/ No
13. Q: Which of the following applies when chewing meals?
    A: Chew well/ Sometimes difficult/Always difficult
14. Q: Do you eat faster than others?
    A: Fast/ Normal/ Slow
15. Q: Do you have dinner within 2 hours before going to bed more than 3 times a week?
    A: Yes/ No

16. Q: Do you take snacks or sweet drinks in addition to the three meals regularly?
    A: Yes/ No
17. Q: Do you skip breakfast more than 3 times a week?
    A: Yes/ No
18. Q: How often do you drink alcohol (such as sake, shochu, beer, whisky etc.)?
    A: Everyday / Sometimes/ None
19. Q: The day you drink, how much alcohol do you consume?
    A: Less than 180ml / 180-360ml/ 360-540ml/ More than 540ml
20. Q: Do you sleep enough?
    A: Yes/ No
21. Q: Do you want to improve your lifestyle (life habit) such as exercise or eating?
    A: I am not planning on improving/ I would like to try/ I am improving (less than 6 months)/ I am improving (more than 6 months)
22. Q: If you have any chance to get a health guidance on improving your life style(life habit), will you use it?
    A: Yes/ No

Supplemental Figure-S1. Cox Proportional Hazard Model of the combination of BMI and lifestyles and the progression in kidney damage among overall subjects.

	n(%)	Hazard Ratio	95% CI		P Value
Smoking					
No smoking with lower BMI	945(43)		reference		
Smoking with lower BMI	285(13)	1.35	0.86	2.09	0.189
No smoking with higher BMI	764(34)	2.15	1.60	2.89	<0.0001
Smoking with higher BMI	213(10)	2.55	1.73	3.76	<0.0001
Alcohol consumption					
Rarely drinking alcohol with lower BMI	605(28)		reference		
Frequent drinking alcohol with lower BMI	619(28)	0.75	0.50	1.12	0.154
Rarely drinking alcohol with higher BMI	510(23)	1.82	1.28	2.58	0.001
Frequent drinking alcohol with higher BMI	462(21)	1.76	1.23	2.51	0.002
Eating snacks between meals					
No snacks with lower BMI	952(43)		reference		
Eating snacks with lower BMI	274(12)	1.66	1.04	2.66	0.033
No snacks with higher BMI	717(33)	2.23	1.68	2.96	<0.0001
Eating snacks with higher BMI	254(12)	2.58	1.72	3.86	<0.0001
Skipping breakfast					
No skipping breakfast with lower BMI	1016(46)		reference		
Skipping breakfast with lower BMI	210(10)	1.07	0.63	1.82	0.796
No skipping breakfast with higher BMI	809(37)	2.11	1.61	2.77	<0.0001
Skipping breakfast with higher BMI	160(7)	2.05	1.30	3.21	0.002
Regular exercise					
Regular exercise with lower BMI	266(12)		reference		
No exercise with lower BMI	964(44)	0.76	0.49	1.18	0.228
No exercise with higher BMI	779(35)	1.76	1.17	2.67	0.007
Regular exercise with higher BMI	197(9)	1.49	0.89	2.48	0.128
Daily walking					
Daily walking with lower BMI	422(19)		reference		
No daily walking with lower BMI	803(36)	0.92	0.61	1.37	0.673
No daily walking with higher BMI	673(31)	2.01	1.39	2.90	0.000
Daily walking with higher BMI	299(14)	1.84	1.20	2.85	0.006
Adequate sleep					
Adequate sleep with lower BMI	808(37)		reference		
No adequate sleep with lower BMI	412(19)	1.11	0.74	1.67	0.628
No adequate sleep with higher BMI	353(16)	2.20	1.54	3.15	<0.0001
Adequate sleep with higher BMI	609(28)	2.14	1.57	2.92	<0.0001

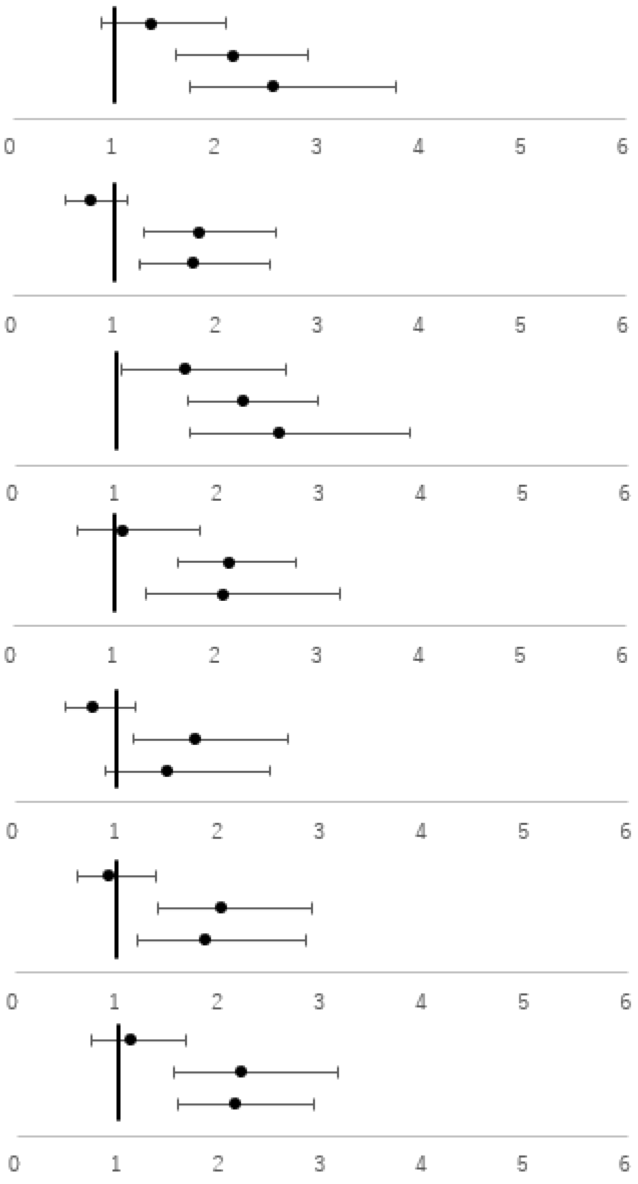

The models were adjusted for age and sex

Supplemental Figure-S2. Cox Proportional Hazard Model of the combination of BMI and lifestyles and the progression in kidney damage with a definition of overweight/obese as BMI≥25 among females.

	n(%)	Hazard Ratio	95%CI		P Value
**Smoking**					
No smoking with BMI<25.0	671(67)	reference			
Smoking with BMI<25.0	58(6)	0.87	0.31	2.40	0.786
No smoking with BMI≥25.0	246(24)	1.74	1.14	2.66	0.010
Smoking with BMI≥25.0	29(3)	0.92	0.22	3.77	0.905
**Alcohol consumption**					
Rarely drinking alcohol with BMI<25.0	512(51)	reference			
Frequent drinking alcohol with BMI<25.0	211(21)	0.49	0.25	0.96	0.039
Rarely drinking alcohol with BMI≥25.0	197(20)	1.30	0.81	2.09	0.283
Frequent drinking alcohol with BMI≥25.0	78(8)	1.75	0.93	3.31	0.082
**Eating snacks between meals**					
No snacks with BMI<25.0	569(57)	reference			
Eating snacks with BMI<25.0	159(16)	1.42	0.75	2.69	0.288
No snacks with BMI≥25.0	192(19)	1.78	1.12	2.83	0.015
Eating snacks with BMI≥25.0	81(8)	1.91	0.93	3.93	0.078
**Skipping breakfast**					
No skipping breakfast with BMI<25.0	639(64)	reference			
Skipping breakfast with BMI<25.0	85(9)	0.91	0.39	2.13	0.824
No skipping breakfast with BMI≥25.0	242(24)	1.68	1.09	2.59	0.019
Skipping breakfast with BMI≥25.0	31(3)	1.62	0.58	4.49	0.354
**Regular exercise**					
Regular exercise with BMI<25.0	135(13)	reference			
No exercise with BMI<25.0	594(59)	0.43	0.25	0.76	0.003
No exercise with BMI≥25.0	227(23)	0.89	0.50	1.58	0.681
Regular exercise with BMI≥25.0	48(5)	0.96	0.42	2.19	0.931
**Daily walking**					
Daily walking with BMI<25.0	228(23)	reference			
No daily walking with BMI<25.0	497(50)	0.92	0.53	1.58	0.753
No daily walking with BMI≥25.0	184(18)	1.70	0.96	3.02	0.069
Daily walking with BMI≥25.0	89(9)	1.19	0.55	2.60	0.660
**Adequate sleep**					
Adequate sleep with BMI<25.0	439(44)	reference			
No adequate sleep with BMI<25.0	284(29)	0.94	0.56	1.59	0.817
No adequate sleep with BMI≥25.0	109(11)	1.20	0.61	2.36	0.605
Adequate sleep with BMI≥25.0	162(16)	1.93	1.17	3.18	0.010

The models were adjusted for age

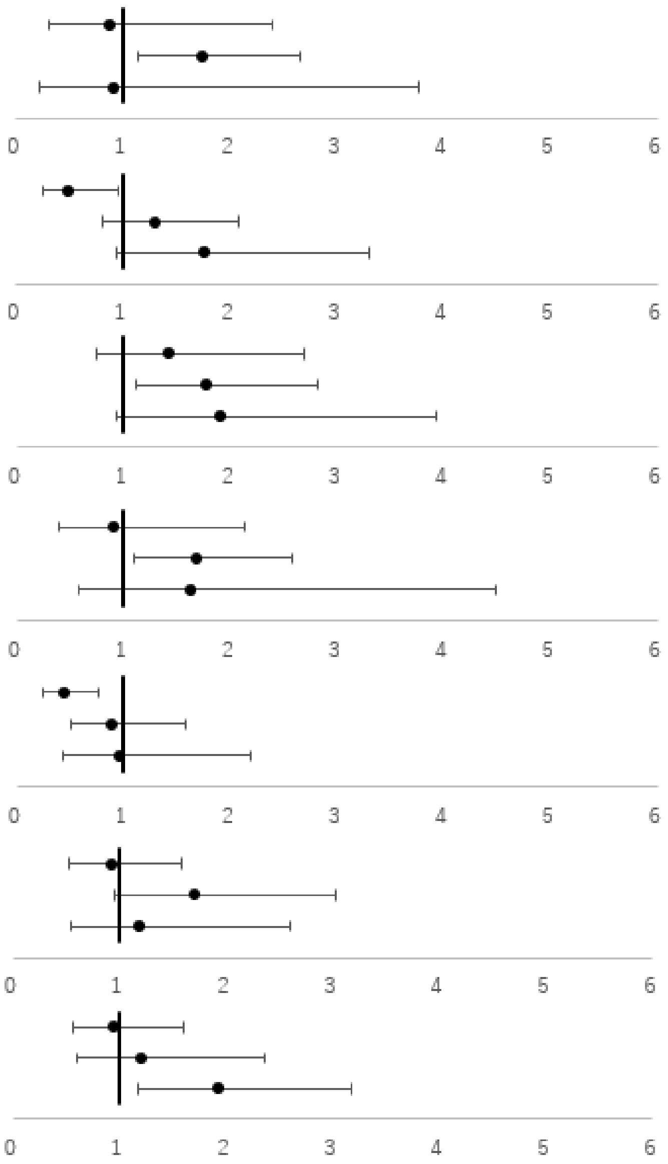

Supplement: Supplementary file 1 [file ijerph-20-05338-s001.zip › ijerph-2274185-supplementary.pdf]
